# Association between Patients’ Self-Judgement, Coagulated Menstrual Blood, and Menorrhagia: Results from a Questionnaire Survey and Blood Test Analysis

**DOI:** 10.3390/medicina59050874

**Published:** 2023-05-01

**Authors:** Eun Ji Lee, Ji Eun Ahn, Jung Min Ryu, Yoon Young Jeong, Youn Seok Choi

**Affiliations:** Department of Obstetrics and Gynecology, School of Medicine, Daegu Catholic University, Daegu 42472, Republic of Korea; unji4243@naver.com (E.J.L.); ahn213@naver.com (J.E.A.); medgirl87@naver.com (J.M.R.)

**Keywords:** menorrhagia, anemia, questionnaire survey, coagulated menstrual blood, self-judgement

## Abstract

*Background and Objectives*: Menorrhagia is defined as a blood loss of more than 80 mL, which is significant enough to cause anemia. Previously known methods for evaluating menorrhagia, such as the alkalin-hematin method, pictograms, and measuring the weight of sanitary products, were all impractical, complex, and time-consuming. Therefore, this study aimed to determine which item among menstrual history taking was most associated with menorrhagia and devised a simple evaluating method for menorrhagia through history taking that can be applied clinically. *Materials and Methods*: The study was conducted from June 2019 to December 2021. A survey was conducted on premenopausal women who underwent outpatient treatment or surgery and those who underwent a gynecologic screening test, and their blood tests were analyzed. The presence of iron deficiency anemia was identified with a Hb level of less than 10 g/dL with microcytic hypochromic anemia on a complete blood count performed within one month of the survey. A questionnaire survey was conducted on six items related to menorrhagia to investigate whether each item was related to “significant menorrhagia”. *Results*: There were 301 participants in the survey during the period. In univariate analysis, the results revealed a statistically significant association between significant menorrhagia and the following items: self-judgement of menorrhagia; menstruation lasting over 7 days; total pad counts in a single menstrual period; Number of sanitary products changed per day; and leakaging of menstrual blood and presence of coagulated menstrual blood. In multivariate analysis, only the “self-judgement of menorrhagia” item showed a statistically significant result (*p*-value = 0.035; an odds ratio = 2.217). When the “self-judgement of menorrhagia” item was excluded, the “passage of clots larger than one inch in diameter” item showed a statistically significant result (*p*-value = 0.023; an odds ratio = 2.113). *Conclusions*: “Patient self-judgement of menorrhagia” is a reliable item for evaluating menorrhagia. Among several symptoms indicating menorrhagia, determining the presence of the “passage of clots larger than one inch in diameter” during the menstrual period is the most useful item for evaluating menorrhagia in clinical history taking. This study suggested using these simple menstrual history taking items to evaluate menorrhagia in real clinical practice.

## 1. Introduction

History taking for menorrhagia is usually performed at gynecological clinics. Menorrhagia is a very important symptom that may be an indication for surgery in various gynecological diseases (leiomyoma, adenomyosis, endometrial glandular hyperplasia, dysfunctional uterine bleeding, etc.) [1,2,3]. Surgery may be considered if there is a significant amount of bleeding due to gynecological diseases; however, accurate measurement of menorrhagia is difficult [4].

A normal menstrual cycle is 24–38 days, and the menstrual period lasts for approximately 4–8 days. The average amount of bleeding is 5 to 80 mL [5,6,7]. According to the known literature, menorrhagia occurs when there is bleeding of 80 mL or more during one menstrual cycle [5]. This is because anemia occurs when the blood loss per menstrual cycle exceeds 80 mL. 

The alkaline–hematin method has traditionally been considered the gold standard for objectively evaluating menstrual blood loss [8,9,10,11,12,13]. This method involves collecting used sanitary products and measuring the alkaline–hematin absorbance of the patient’s menstrual blood. However, it can be expensive and burdensome for patients to collect and store sanitary products, and it does not account for external menstrual blood loss. These disadvantages limit its usefulness in determining the amount of blood loss due to menorrhagia in clinical practice. 

Other conventional methods for measuring menstrual blood loss include the use of pictograms and weighing sanitary products [12,13,14,15,16,17,18,19,20,21,22]. The menstrual pictogram is a relatively accurate and sensitive test that provides a score for each sanitary product. This method involves scoring each sanitary product and using the total score to measure menstrual blood loss volume, which is simple and quick. However, the results must be recorded each time the patient changes sanitary products. Additionally, since the physiological amount of blood loss may vary with each menstrual cycle, there can be great fluctuations in scores with each cycle, limiting its applicability. As for the sanitary product weighing method, it is difficult to accurately measure the amount of blood loss because it does not account for external blood loss, such as the amount of blood shed during showering or urination [23]. In the end, there are limitations to easily applying any of these methods in actual clinical practice.

From the patient’s point of view, it is difficult to determine whether menstrual flow is excessive because they have not compared their menstrual blood loss volume with that of others. In fact, clinicians often ask patients with gynecological conditions whether they have heavy menstrual bleeding during menstruation. In addition, in most cases, the question is asked without specific criteria about menorrhagia. Therefore, many patients are often unable to give a clear answer [24,25].

According to the literature, the following items can be evaluated during history taking for menorrhagia [26]: changing of pads or tampons more often than every 3 h, use of more than 20 pads during a single menstruation period, the need to change pads during the night, frequent episodes of accidental soiling of clothing and bedsheets, passage of clots greater than 1 inch in diameter, menses lasting longer than 7 days, and a diagnosis of anemia. However, which of these items is most associated with menorrhagia remains unknown.

Therefore, this study aimed to determine which item among the menstrual history taking questionnaire was most associated with menorrhagia accompanied by anemia. In addition, we tried to devise a simple evaluation method for menorrhagia through history taking that can be applied clinically.

## 2. Materials and Methods

Type of study: From June 2019 to December 2021, a prospective survey was conducted using a questionnaire survey and blood test analysis among participants. 

Inclusion criteria: Premenopausal women who underwent outpatient treatment or surgery at the Daegu Catholic University Medical Center and those who underwent a gynecologic screening test. 

Exclusion criteria: “Pregnant women and women with an intrauterine device in situ, women without a uterus after hysterectomy, malignant diseases, infectious diseases, or coagulopathy diseases” that could affect the study results were excluded from the study. 

The presence of iron deficiency anemia was identified based on the hemoglobin (Hb) level, mean corpuscular volume (MCV), and the mean corpuscular hemoglobin concentration (MCHC) on a complete blood count (CBC) performed within one month of the survey. In this study, “significant menorrhagia” was defined as moderate anemia with a Hb level of less than 10 g/dL [27] with microcytic hypochromic anemia or iron deficiency anemia with iron supplementation [28]. Patients with anemia from other causes, such as vitamin B12 deficiency or aplastic anemia, were excluded.

Our questionnaire survey was administered to the participants in paper form by using Appendix A. Our questionnaire survey included the questions in Table 1. In this study, ‘a diagnosis of anemia’ item was set as a dependent variable; therefore, the questionnaire was composed of items presented in the literature excluding this item. Instead of this item, we added “self-judgement of menorrhagia” as the first question. 

A questionnaire survey was conducted on these 6 items to investigate whether each item was related to “significant menorrhagia”. There were six major categories of items, and each category was divided into questions that set detailed standards and questions that could be answered subjectively (questions 2, 3, and 4).

In this study, univariate and multivariate analyses were performed on whether each item was associated with “significant menorrhagia” that is defined in our study.

Statistical analyses were performed using IBM SPSS statistics V25.0 (IBM, Armonk, NY, USA). Comparison of categorical variables was performed using the chi-squared test. Comparison of the mean values for continuous variables was performed using an independent t-test. Multivariable analysis was performed using logistic regression. *p*-values were the result of a two-sided test, and a *p* < 0.05 was considered statistically significant.

This prospective study was approved by the Ethics Committee of our institution (CR-22-011-L). All procedures performed involving human participants were in accordance with the ethical standards of our Institutional Research Committee, National Research Committee, and the 1964 Helsinki Declaration and its later amendments, or with comparable ethical standards.

## 3. Results

There were 301 participants in the survey during the survey period. Among the 301 patients, 211 were diagnosed with benign gynecological diseases. The distribution of the patients is shown in Table 2. According to the survey results, the largest number of benign gynecological diseases reported was leiomyoma (n = 63), followed by benign ovarian cysts (n = 47) and cervical intraepithelial neoplasia (n = 37). Approximately 90 participants did not have any confirmed benign gynecological disease.

The results of the univariate analysis on the association between each item of the menstrual history taking questionnaire and significant menorrhagia are shown in Table 3. The analysis revealed a statistically significant association between significant menorrhagia and the items “self-judgement of menorrhagia” (*p*-value < 0.001); “menses lasting longer than 7 days” (*p*-value = 0.008); “total pad counts in a single menstrual period” (*p*-value = 0.001); “number of pads on the day with the heaviest menstrual flow (*p*-value = 0.023); “period of changing pads or tampons more often than 3 h” (*p*-value = 0.002); “leakaging of menstrual blood” (*p*-value = 0.005); and “presence of coagulated menstrual blood” (*p*-value <0.001). There was no statistically significant association between “use of more than 20 pads during a single menstrual period” and significant menorrhagia.

**Figure 1:**
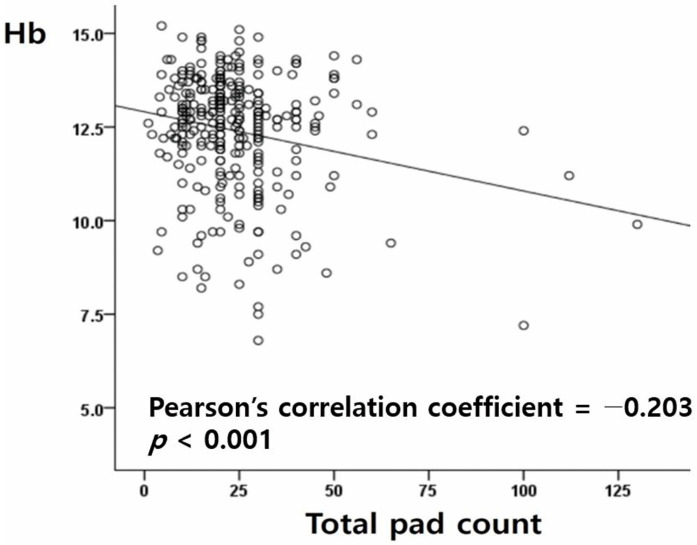
Correlation between serum hemoglobin level and total pad count.

There was a statistically significant association between significant menorrhagia and the total number of pads used during a menstrual period. The correlation between Hb level and the total pad count is shown in Figure 1. It was confirmed that the higher the total number of pads, the lower the Hb level (Pearson’s correlation coefficient = −0.203; *p* < 0.001).

**Figure 2:**
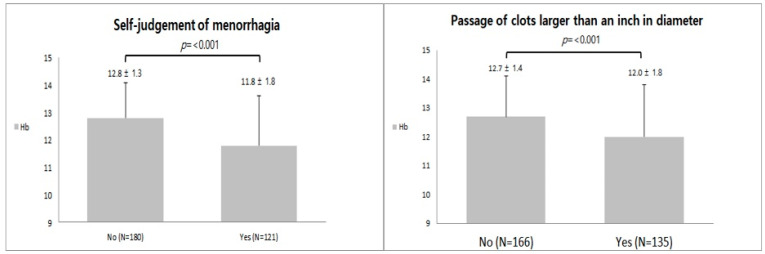
Differences in serum hemoglobin level according to “self-judgement of menorrhagia” and “passage of clots larger than one inch in diameter”.

There was a statistically significant difference in Hb level according to “self-judgement of menorrhagia” (*p* < 0.001). There was a difference of approximately 1 g/dL in the average Hb level between the group that answered yes to “self-judgment of menorrhagia” and the group that answered no. Additionally, there was a statistically significant difference in hemoglobin level based on the response to “passage of clots larger than one inch in diameter” (*p* < 0.001), with a difference of approximately 0.7 g/dL in the average hemoglobin level between the group that answered yes and the group that answered no (Figure 2).

The items that were statistically significant in the univariate analysis were extracted and used in performing the multivariate analysis. In the multivariate analysis, each statistical result was analyzed according to the “self-judgement of menorrhagia” item, which was included to account for cases in which patients could not judge their menstrual flow by themselves. As shown in Table 4, only the “self-judgement of menorrhagia” item showed a statistically significant result (*p*-value = 0.035; an odds ratio = 2.217) when it was included. The remaining items did not show statistical significance. When the “self-judgement of menorrhagia” item was not included, the most significant result was obtained for the item “passage of clots larger than one inch in diameter”, with a *p*-value of 0.023 and an odds ratio of 2.113 (shown in Table 5).

## 4. Discussion

Anemia is a medical condition in which the number of red blood cells or the amount of hemoglobin in the blood is below normal, leading to reduced oxygen delivery to the body’s tissues. It can also be caused by a lack of certain vitamins or minerals that are essential for red blood cell production, such as iron, vitamin B12, or folic acid. Anemia is defined as a Hb level of less than 12 g/dL for mild anemia (normal Hb level is 12–16 g/dL [women]), and less than 10 g/dL for moderate anemia.

Iron deficiency anemia in non-pregnant female adults is defined as ferritin < 15 micrograms/L, hemoglobin < 12 g/dL, and microcytic, hypochromic red blood cells on the CBC test. Menorrhagia is one of the major risk factors for iron deficiency anemia (IDA). It can lead to depletion of iron stores, particularly in women, as the amount of blood lost during heavy menstrual bleeding increases iron consumption. This can result in iron deficiency anemia, where there is not enough iron available to make sufficient hemoglobin in the body. Menorrhagia with iron deficiency anemia can also lead to other complications such as fatigue, weakness, shortness of breath, and decreased exercise tolerance, which can further affect a woman’s quality of life [29,30,31].

Menorrhagia is defined as a blood loss of more than 80 mL, which is significant enough to cause anemia [5,32]. There are various treatment methods for menorrhagia. The following are some commonly used methods: (1) Medication: For mild menorrhagia, obesity-related or hormonal medication can be attempted. For obesity-related menorrhagia, oral contraceptives or an intrauterine hormone-releasing device (IUD) can be used. For hormonal menorrhagia, progesterone, synthetic estrogen, synthetic progesterone, or a combination of both estrogen and progesterone can be used. (2) Surgery: Surgery may be necessary for moderate to severe menorrhagia. Surgical treatments include hysterectomy, uterine artery embolization, endometrial ablation, etc. It is important to determine the appropriate treatment method through appropriate diagnosis and examination before surgery.

The treatment method varies depending on the cause of the patient’s menorrhagia, age, health status, and treatment intention. Therefore, it is important to consult with a specialist after appropriate diagnosis and examination to determine the appropriate treatment method.

Although we are aware of the importance of evaluating menorrhagia, in actual clinical practice, the exact amount of blood loss per cycle in women with complaints of menorrhagia cannot be accurately measured. Conventionally known methods for objectively assessing menorrhagia include the alkaline–hematin method, the use of pictograms, and the weighing of sanitary products [33,34]. 

The alkaline–hematin method is considered the gold standard for objective measurement of menstrual blood loss volume [35]. However, it is expensive and inconvenient for patients to collect and store sanitary products, and it does not account for external menstrual blood loss. The menstrual pictogram is a relatively accurate and sensitive test that assigns a score to sanitary products [13,36,37]. The method of scoring each sanitary product and using the total score to measure menstrual blood loss volume is simple and quick, but results must be recorded each time the patient changes sanitary products. The sanitary product weighing method has a high possibility of omitting some measurements because the patient must weigh the sanitary product every time it is changed [14,15,23,38].

While these previously studied methods may be quantitative and academic, these methods for evaluating menorrhagia are difficult to apply in actual outpatient settings; therefore, our study aimed to find a simple method for evaluating menorrhagia that can be used in real outpatient clinics.

Therefore, we selected six items related to menstrual history taking mentioned in the literature, which are easy to apply clinically, to determine which item among the menstrual history taking questionnaire was most associated with menorrhagia accompanied by anemia [26]. Statistical analysis of the six items of the menstrual history taking questionnaire revealed that each item was associated with a clinically significant menorrhagia: “self-judgement of menorrhagia; lasting days of menstruation over 7 days; total pad counts in a single menstrual period; number of changing sanitary product on the day; leakaging of menstrual blood and presence of coagulated menstrual blood”. Among these items, as shown by Figure 1, it was found that the item “total pad counts in a single menstrual period” is associated with hemoglobin level. Through these results, it was confirmed that most of the items presented by Speroff’s textbook had clinical significance. Especially, apart from the history taking items suggested in the literature, we also tried to verify “self-judgment of menorrhagia”. “Self-judgement of menorrhagia” is commonly misunderstood to be a subjective factor; however, it was found to be the item among the six items that was most significantly associated with significant menorrhagia (*p*-value = 0.035; an odds ratio = 2.217). It is surprising that, in the multivariate analysis including self-judgment of menorrhagia, none of the other items showed statistical significance. It was unexpected that self-judgment of menorrhagia, which is the most subjective item, also showed a strong correlation. This could mean that patients themselves judge whether their menstrual flow is excessive based on various factors. Therefore, it is important to ask about “self-judgement of menorrhagia” to determine menorrhagia when taking a gynecological history.

However, if it is difficult for a patient to determine menorrhagia herself, it is clinically helpful to ask the patient about the other five items of the menstrual history taking questionnaire to evaluate menorrhagia. Among these five items, “the passage of clots larger than one inch in diameter” was the most significant item (*p*-value = 0.023; an odds ratio = 2.113). Among our survey items, “total pad counts during a single menstrual period” and “changing pads more often than every 3 h” may be less reliable than other items because patients that have a sensitive personality or frequently change their sanitary pads, even when the pads are slightly wet, may change their pads more frequently; therefore, the number of pads may not accurately reflect the volume of their menses. In addition, through Figure 2, we have confirmed that the two items of “self-judgment of menorrhagia” and “the passage of clots larger than one inch in diameter” are statistically associated with Hb (*p*-value < 0.001), reaffirming that these history taking items have a direct numerical correlation with menstrual blood loss.

Pamela E. Warner et al. identified the features of clinical history that best predict menorrhagic blood loss. Similar with our study results, this study also found that a woman’s self-judgement of menorrhagia was related to the measured blood loss, contradicting the clinical belief that women are poor judges of their menstrual blood loss volume. However, in that study, subjective judgement of their period heaviness was not compared to other clinical history items. In our study, taking this into account, we were able to confirm once again that self-judgement of menorrhagia produces the most significant results even when compared to other clinical history items [32].

Regarding “the passage of clots larger than an inch in diameter” item, the formation of blood clots in menorrhagia is related to the proliferation and shedding of the endometrium. During the menstrual cycle, the endometrium thickens and the blood vessels that supply blood to it increase in number. Towards the end of the menstrual cycle, the endometrium sheds and these blood vessels rupture, causing bleeding. If the blood is unable to leave the site of bleeding quickly and remains in the endometrium for a long time, it can coagulate and form a blood clot. Blood clots can cause the endometrium to detach and if they are large or numerous, they can lead to menorrhagia [1]. Interestingly, in another study on clotting of the menstrual blood, pathomorphological and biochemical analysis of menstrual blood clots revealed that these clots are not composed of fibrin. They are not products of coagulation but are red cell aggregations on mucoid substances, mucoproteins, and/or glycogen [39]. Therefore, the blood clots larger than one inch may be associated with menorrhagia with anemia because these blood clots are products of red blood cell aggregation. Based on these principles, the association between blood clots and menorrhagia can be explained.

Through this study, we were able to confirm the importance of “self-judgement of menorrhagia” and “the passage of clots larger than an inch in diameter” in surveys regarding symptoms associated with menorrhagia.

Our study has some limitations. First, this study is a single-center study. Second, when identifying IDA, ferritin levels were not routinely measured in all participants’ blood test analysis. However, this study is a prospective study. Despite being a single-center study, a considerable number of participants were enrolled.In addition, this study would be helpful in the cost-effective evaluation of menorrhagia.

## 5. Conclusions

In clinical practice, menstrual history taking items can be used efficiently to evaluate menorrhagia. Among these items, “self-judgement of menorrhagia” is sufficiently reliable for assessing menorrhagia. In addition, among various symptoms suggesting menorrhagia, determining whether there is a “passage of blood clots larger than one inch in diameter” can be most useful when evaluating menorrhagia among the items for menstrual history taking. This study suggested using these simple menstrual history taking items as a more systematic tool to evaluate menorrhagia in real clinical practice, instead of relying on subjective questions.

## Figures and Tables

**Table 1:** Questions about menstrual history taking.

1. Self-judgement: do you believe that you have an excessive menstrual flow?
2. Lasting days: - Does your menses last for more than 7 days? - Total number of days in a single menstrual period 3. Total pad counts per single menstrual period: - Do you use more than 20 pads during a single menstrual period? - Total pad counts in a single menstrual period 4. Number of sanitary products changed per day: - Do you need to change pads or tampons more frequently than every 3 h? - Number of pads on the day with the heaviest menstrual flow 5. Leakaging of menstrual blood: do you experience frequent episodes of accidental soiling of your clothing or bedsheet? 6. Presence of coagulated menstrual blood: do you pass blood clots that are larger than one inch in diameter?

**Table 2:** Number of women with or without gynecologic disease.

Women with Gynecological Diseases	Number
Myoma	63
Myoma with benign ovarian cyst	11
Adenomyosis	9
Adenomyosis with benign ovarian cyst	6
Myoma with Adenomyosis	14
EM pathology (EM polyp, EM hyperplasia, etc.)	15
Benign ovarian cyst	47
CIN~CIS	37
TOA	2
Others	7
Women without known gynecological diseases	90
Total	301

Abbreviation: EM: endometrial, CIN: cervical intraepithelial neoplasia, CIS: carcinoma in situ, and TOA: tubo-ovarian abscess.

**Table 3:** Univariate analysis of the association between “significant menorrhagia” and the items of the menstrual history questionnaire.

	No. (%) Or Mean ± SD	Odds Ratio (95% CI)	*p*-Value
-Self-judgement of menorrhagia	121 (40.2%)	3.818 (2.133–6.835)	<0.001 *
-Total number of days in a single menstrual period (days, mean ± SD)	6.0 ± 3.4	1.069 (0.994–1.150)	0.074
-Menses lasting longer than 7 days	26 (8.6%)	3.072 (1.334–7.071)	0.008 *
-Total pad counts in a single menstrual period (counts, mean ± SD)	24.1 ± 15.3	1.033 (1.014–1.052)	0.001 *
-Use of 20 or more pads per menstrual cycle	198 (63.8%)	1.740 (0.932–3.249)	0.082
-Number of pads on the day with the heaviest menstrual flow (counts, mean ± SD)	6.2 ± 3.6 (296/301)	1.086 (1.011–1.167)	0.023 *
-Changing of pads more often than every 3 h	63 (21.1%) (298/301)	2.616 (1.408–4.861)	0.002 *
-Accidental leakage of menses on bed sheets	164 (54.5%)	2.349 (1.297–4.252)	0.005 *
-Passage of clots larger than one inch in diameter	135 (44.9%)	3.205 (1.778–5.745)	<0.001 *

Abbreviation: SD: standard deviation, CI: confidence interval, *: statistically significant difference, and significant menorrhagia = heavy menstrual flow sufficient to cause iron deficiency anemia.

**Table 4:** Multivariate analysis of the association between “significant menorrhagia” and the items of the menstrual history questionnaire including self-judgement of menorrhagia.

	Odds Ratio	95% CI	*p*-Value
Self-judgement of menorrhagia	2.217	1.057–4.647	0.035
Menses lasting longer than 7 days	1.638	0.625–4.288	0.315
Total pad counts in a single menstrual period	1.011	0.989–1.034	0.316
Changing of pads more often than every 3 h	1.262	0.588–2.706	0.550
Leakaging of menstrual blood	1.011	0.484–2.113	0.976
Presence of coagulated menstrual blood	1.886	0.977–3.642	0.059

Abbreviation: CI: confidence interval and significant menorrhagia = heavy menstrual flow sufficient to cause iron deficiency anemia.

**Table 5:** Multivariate analysis of the association between “significant menorrhagia” and the items of the menstrual history questionnaire excluding self-judgement of menorrhagia.

	Odds Ratio	95% CI	*p*-Value
Menses lasting longer than 7 days	1.867	0.721–4.837	0.199
Total pad counts in a single menstrual period	1.014	0.992–1.036	0.226
Changing of pads more often than every 3 h	1.422	0.669–3.022	0.359
Leakaging of menstrual blood	1.413	0.737–2.712	0.298
Presence of coagulated menstrual blood	2.113	1.111–4.019	0.023

Abbreviation: CI: confidence interval and significant menorrhagia = heavy menstrual flow sufficient to cause iron deficiency anemia.

## Data Availability

Data for this study, though not available in a public repository, will be made available to other researchers upon reasonable request.

## Appendix A

Menorrhagia history taking questionnaire

Name	
Date of birth	Year month date
Patient’s number	

Please answer the symptoms related to excessive menstrual flow.

1. Do you believe that you have an excessive menstrual flow?           □ Yes           □ No
2. Is your menstrual cycle regular?cycles regular?                 □ Regular                 □ Irregular
3. How many days is your total menstrual period? How many days do you have heavy bleeding during your period?
  - Total menstrual period                       days
  - Heavy bleeding days during menstruation                       days
4. How many total sanitary pads (or tampons) do you use during your menstrual period?
  - Total                       
5. How many sanitary pads (or tampons) do you use per day on heavy menstrual days?
  - Use of sanitary pads (or tampons) on heavy                       days
6. Will your clothes get wet if you don’t use large sanitary napkins, such as overnight sanitary napkins, or multiple sanitary pads in layers during the night?            □ Yes             □ No
7. Do you pass blood clots that are larger than one inch in diameter during your menstrual period?          □ Yes          □ No
8. Have you ever taken an iron supplement or received an iron supplement injection within the last few months due to anemia?          □ Yes          □ No
9. Are you using contraceptives, intrauterine devices (such as Mirena), and implantable contraceptives (such as Implanon) due to excessive menstrual flow?          □ Yes          □ No
10. Are you taking anticoagulants (warfarin, herapin, etc.) or antiplatelet/antithrombotic drugs (Aspirin, plavix or any other antiplatelet /antithrombotic drugs)?          □ Yes          □ No
11. Do you have a coagulopathy disorder or bleeding disorder (thrombocytopenia, leukemia, etc.)?          □ Yes          □ No

## References

[1] Apgar B.S., Kaufman A.H., George-Nwogu U., Kittendorf A.L. (2007). Treatment of menorrhagia. Am. Fam. Physician.

[2] Oehler M.K., Rees M.C. (2003). Menorrhagia: An update. Acta Obstet. Gynecol. Scand..

[3] Donnez J. (2011). Menometrorrhagia during the premenopause: An overview. Gynecol. Endocrinol..

[4] Berek J.S., Berek D.L. (2020). Berek & Novak’s Gynecology.

[5] Hallberg L., Högdahl A.-M., Nilsson L., Rybo G. (1966). Menstrual blood loss—A population study. Variation at different ages and attempts to define normality. Acta Obstet. Gynecol. Scand..

[6] Warner P.E., Critchley H.O., Lumsden M.A., Campbell-Brown M., Douglas A., Murray G.D. (2004). Menorrhagia II: Is the 80-mL blood loss criterion useful in management of complaint of menorrhagia?. Am. J. Obstet. Gynecol..

[7] Walker M.H., Coffey W., Borger J. (2023). Menorrhagia. StatPearls.

[8] Fraser I.S., Warner P., Marantos P.A. (2001). Estimating menstrual blood loss in women with normal and excessive menstrual fluid volume. Obstet. Gynecol..

[9] van Eijkeren M.A., Scholten P.C., Christiaens G.C., Alsbach G.P., Haspels A.A. (1986). The alkaline hematin method for measuring menstrual blood loss—A modification and its clinical use in menorrhagia. Eur. J. Obstet. Gynecol. Reprod. Biol..

[10] Magnay J.L., Schönicke G., Nevatte T.M., O’Brien S., Junge W. (2011). Validation of a rapid alkaline hematin technique to measure menstrual blood loss on feminine towels containing superabsorbent polymers. Fertil. Steril..

[11] Magnay J.L., Nevatte T.M., Dhingra V., O’Brien S. (2010). Menstrual blood loss measurement: Validation of the alkaline hematin technique for feminine hygiene products containing superabsorbent polymers. Fertil. Steril..

[12] Haberland C., Filonenko A., Seitz C., Börner M., Gerlinger C., Doll H., Wessiepe D. (2020). Validation of a menstrual pictogram and a daily bleeding diary for assessment of uterine fibroid treatment efficacy in clinical studies. J. Patient Rep. Outcomes.

[13] Ko J.K.Y., Lao T.T., Cheung V.Y.T. (2021). Pictorial Blood Loss Assessment Chart for evaluating heavy menstrual bleeding in Asian women. Hong Kong Med. J..

[14] Magnay J.L., O’Brien S., Gerlinger C., Seitz C. (2018). A systematic review of methods to measure menstrual blood loss. BMC Womens Health.

[15] Magnay J.L., Nevatte T.M., O’Brien S., Gerlinger C., Seitz C. (2014). Validation of a new menstrual pictogram (superabsorbent polymer-c version) for use with ultraslim towels that contain superabsorbent polymers. Fertil. Steril..

[16] Magnay J.L., O’brien S., Gerlinger C., Seitz C. (2020). Pictorial methods to assess heavy menstrual bleeding in research and clinical practice: A systematic literature review. BMC Womens Health.

[17] Schumacher U., Schumacher J., Mellinger U., Gerlinger C., Wienke A., Endrikat J. (2012). Estimation of menstrual blood loss volume based on menstrual diary and laboratory data. BMC Womens Health.

[18] Fraser I.S., Zeun S., Parke S., Wilke B., Junge W., Serrani M. (2013). Improving the objective quality of large-scale clinical trials for women with heavy menstrual bleeding: Experience from 2 multi-center, randomized trials. Reprod. Sci..

[19] Matteson K.A. (2017). Menstrual questionnaires for clinical and research use. Best. Pract. Res. Clin. Obstet. Gynaecol..

[20] Liberty A., Samuelson Bannow B., Matteson K., Edelman A., Colwill A. (2023). Menstrual Technology Innovations and the Implications for Heavy Menstrual Bleeding. Obstet. Gynecol.

[21] Toxqui L., Pérez-Granados A.M., Blanco-Rojo R., Wright I., Vaquero M.P. (2014). A simple and feasible questionnaire to estimate menstrual blood loss: Relationship with hematological and gynecological parameters in young women. BMC Womens Health.

[22] Perelló J., Pujol P., Pérez M., Artés M., Calaf J. (2022). Heavy Menstrual Bleeding-Visual Analog Scale, an Easy-to-Use Tool for Excessive Menstrual Blood Loss That Interferes with Quality-of-Life Screening in Clinical Practice. Womens Health Rep..

[23] Ruta D.A., Garratt A.M., Chadha Y.C., Flett G.M., Hall M.H., Russell I.T. (1995). Assessment of patients with menorrhagia: How valid is a structured clinical history as a measure of health status?. Qual. Life Res..

[24] Dutton B., Kai J. (2023). Women’s experiences of heavy menstrual bleeding and medical treatment: A qualitative study in primary care. Br. J. Gen. Pract..

[25] Lancastle D., Kallner H.K., Hale G., Wood B., Ashcroft L., Driscoll H. (2023). Development of a brief menstrual quality of life measure for women with heavy menstrual bleeding. BMC Womens Health.

[26] Taylor H.S., Pal L., Sell E. (2020). Speroff’s Clinical Gynecologic Endocrinology and Infertility.

[27] Jameson J.L., Kasper D.L., Longo D.L., Fauci A.S., Hauser S.L., Loscalzo J. (2018). Harrison’s Principles of Internal Medicine.

[28] Mansour D., Hofmann A., Gemzell-Danielsson K. (2021). A Review of Clinical Guidelines on the Management of Iron Deficiency and Iron-Deficiency Anemia in Women with Heavy Menstrual Bleeding. Adv. Ther..

[29] Munro M.G., Mast A.E., Powers J.M., Kouides P.A., O’brien S.H., Richards T., Lavin M., Levy B.S. The relationship between heavy menstrual bleeding, iron deficiency, and iron deficiency anemia. Am. J. Obstet. Gynecol..

[30] Cooke A.G., McCavit T.L., Buchanan G.R., Powers J.M. (2017). Iron Deficiency Anemia in Adolescents Who Present with Heavy Menstrual Bleeding. J. Pediatr. Adolesc. Gynecol..

[31] Kawabata H., Tamura T., Tamai S., Fujibayashi A., Sugimura M. (2022). Intravenous ferric derisomaltose versus saccharated ferric oxide for iron deficiency anemia associ-ated with menorrhagia: A randomized, open-label, active-controlled, noninferiority study. Int. J. Hematol..

[32] Warner P.E., Critchley H.O., Lumsden M.A., Campbell-Brown M., Douglas A., Murray G.D. (2004). Menorrhagia I: Measured blood loss, clinical features, and outcome in women with heavy periods: A survey with follow-up data. Am. J. Obstet. Gynecol..

[33] Donoso M.B., Serra R., Rice G.E., Gana M.T., Rojas C., Khoury M., Arraztoa J.A., Monteiro L.J., Acuña S., Illanes S.E. (2019). Normality Ranges of Menstrual Fluid Volume During Reproductive Life Using Direct Quantification of Menses with Vaginal Cups. Gynecol. Obstet. Invest..

[34] Gudmundsdottir B.R., Hjaltalin E.F., Bragadottir G., Hauksson A., Geirsson R.T., Onundarson P.T. (2009). Quantification of menstrual flow by weighing protective pads in women with normal, decreased or increased menstruation. Acta Obstet. Gynecol. Scand..

[35] Shaw S.T., Aaronson D.E., Moyer D.L. (1972). Quantitation of menstrual blood loss--further evaluation of the alkaline hematin method. Contraception.

[36] Singh S.S., Alsina J.C., Vannuccini S., Koga K., Silva-Filho A.L., Yang X., Estrade J.-P., Catherino W. (2022). Clinical perspectives on the menstrual pictogram for the assessment of heavy menstrual bleeding. Hum. Reprod. Open..

[37] Zakherah M.S., Sayed G.H., El-Nashar S.A., Shaaban M.M. (2011). Pictorial blood loss assessment chart in the evaluation of heavy menstrual bleeding: Diagnostic accuracy compared to alkaline hematin. Gynecol. Obstet. Invest..

[38] Quinn S.D., Higham J. (2016). Outcome measures for heavy menstrual bleeding. Womens Health.

[39] Beller F.K. (1971). Observations on the clotting of menstrual blood and clot formation. Am. J. Obstet. Gynecol..

